# The effect of type-2 diabetes conditions on neutrophil rolling adhesion

**DOI:** 10.1186/s13104-022-06248-0

**Published:** 2022-12-03

**Authors:** Keith Taverner, Yousif Murad, Adam B. Yasunaga, Christine Furrer, Jonathan Little, Isaac T. S. Li

**Affiliations:** 1 Department of Chemistry, University of British Columbia Okanagan, 3247 University Way, Kelowna, BC V1V 1V7 Canada; 2 Faculty of Medicine, University of British Columbia, Kelowna, BC Canada; 3 School of Health and Exercise Sciences, University of British Columbia, Kelowna, BC Canada

**Keywords:** HL-60, Neutrophils, Type-2 diabetes mellitus, Cell rolling adhesion, P-selectin, PSGL-1

## Abstract

**Objective:**

Type 2 diabetes mellitus (T2D) is the result of a dysregulation of insulin production and signalling, leading to an increase in both glucose concentration and pro-inflammatory cytokines such as interleukin (IL)-6 and tumour necrosis factor (TNF)-α. Previous work showed that T2D patients exhibited immune dysfunction associated with increased adhesion molecule expression on endothelial cell surfaces, accompanied by decreased neutrophil rolling velocity on the endothelial cell surface. Changes in cell rolling adhesion have direct vascular and immune complications such as atherosclerosis and reduced healing time in T2D patients. While previous studies focused primarily on how endothelial cells affect neutrophil rolling under T2D conditions, little is known about changes to neutrophils that affect their rolling. In this study, we aim to show how the rolling behaviour of neutrophils is affected by T2D conditions on a controlled substrate.

**Results:**

We found that neutrophils cultured in T2D-serum mimicking media increased cell rolling velocity compared to neutrophils under normal conditions. Specifically, glucose alone is responsible for higher rolling velocity. While cytokines further increase the rolling velocity, they also reduce the cell size. Both glucose and cytokines likely reduce the function of P-selectin Glycoprotein Ligand-1 (PSGL-1) on neutrophils.

## Introduction

Neutrophils are first responders in an inflammatory response. They are captured near the inflammatory site via rolling adhesion on the endothelial cell wall before transmigrating out of the bloodstream [1]. This adhesion is mediated by the rapid formation and dissociation of interactions between P-selectin glycoprotein ligand 1 (PSGL-1) on neutrophil membranes, and P-selectin expressed on endothelial surfaces under inflammatory conditions [1, 2]. This tightly regulated process is essential for acute immune responses. Therefore, any changes in rolling adhesion can have profound effects on the immune system.

T2D is associated with high basal cytokine levels and a wide range of innate immune responses [3]. Hyperglycemia has been linked to a pro-inflammatory state leading to increased production of interleukin-6 (IL-6) and tumour necrosis factor α (TNF-α) [4, 5]. Hyperglycemia can also cause insulin resistance from chronic exposure to glucose and reactive oxygen species formation [4, 5]. Patients with T2D can experience complications such as cardiovascular disease, atherosclerosis, blindness, and kidney failure [6, 7]. Atherosclerosis is associated with chronic inflammation, possibly due to the increased recruitment of immune cells, including neutrophils [8].

Previous studies have shown that patients with T2D exhibit immune dysfunction related to neutrophil adhesion [6, 9, 10]. Specifically, PMNs (polymorphonuclear neutrophils) taken from T2D patients have a decreased cell rolling velocity than a healthy control population [6, 10]. These studies examined cell rolling on an activated human umbilical vein endothelial cells (HUVEC) surface where variable concentrations of P-selectin and other adhesion molecules are present [6, 8, 10]. This surface receptor heterogeneity and expression levels significantly affect cell rolling behaviour under T2D conditions [8]. Additionally, these studies used a singular wall shear stress value of 0.07 Pa which may not be sufficient to demonstrate the full range of effects imparted by diabetic growth conditions [6, 10]. Neutrophils roll on this complex surface primarily via interaction with PSGL-1 expressed on their surface. PSGL-1 is a crucial ligand for rolling adhesion and a receptor to enable intracellular signalling, inducing cytokine secretion, activation of SRC family kinase, β2-integrin, and potential activation of Phosphoinositide 3-kinase (PI3K) signalling in neutrophils [11].

This paper aimed to understand how the rolling of neutrophils is affected by T2D conditions, including hyperglycemia and supraphysiological concentrations of TNF-α, IL-6, and insulin. We investigated neutrophil rolling on a surface with a controlled P-selectin density to elucidate the effect of T2D conditions, specifically on the PSGL-1 and P-selectin interactions.

## Main Text

### Methods and materials

#### Flow chamber construction

The flow chamber construction has been reported previously [12–14]. Briefly, the flow chamber consisted of double-sided tape with channels cut into it sandwiched between a coverslip and a glass slide. The surface was passivated by polyethylene glycol (PEG) and functionalized by PEG-biotin (Laysan Bio) at a 20:1 ratio. The biotin moieties were used to anchor P-selectin to the coverslip by the sequential addition of 1 mg/mL streptavidin (Thermofisher), 100 µg/mL biotinylated protein G (Thermofisher) and 10.6 µg/mL P-selectin-Fc (R&D System).

#### Cell growth and rolling experiment

HL-60 cells were cultured in 25 cm^2^ culture flasks (VWR) containing glucose-free RPMI-1640 media (Thermofisher), 10% FBS (VWR), and 1% penicillin–streptomycin (Gibco). Cell cultures were maintained in 5% CO_2_ at 37 °C. HL-60 cells were differentiated into neutrophils using 1.5% dimethyl sulfoxide (DMSO). To make up the final concentrations of pro-inflammatory conditions, 40% glucose (VWR), 1000 pM insulin (Humulin), 10 ng/mL TNF-α (PeproTech), and 10 ng/mL IL-6 (PeproTech) were used. Glucose concentrations were measured using a commercial glucose monitor (Contour) [15]. For chronic exposure, cells were cultured for ~ 72 h under their stated growth media conditions (glucose and pro-inflammatory factors). 2 mL of cell culture was centrifuged for 3.5 min at 300 rcf. Approximately 1.7 mL of liquid was removed, and cells were resuspended in the rolling buffer and used immediately [12]. For acute exposure, cells from cell culture media were washed and resuspended in the rolling buffer with different glucose concentrations and used immediately (30 min, including the rolling experiment). Darkfield microscopy was used to capture cell rolling movies at 30 fps. A syringe pump was used to control the flow rate. Flow rates between 0.5 and 15 mL/hour were used in the experiments, corresponding to wall shear stresses of 0.17 and 1.24 Pa. The observation time was set to allow cells to cross the entire field of view to maximize data acquisition. Videos were analyzed using custom code to quantify cell rolling velocity and size as previously described [13]. Statistical analysis was performed using MATLAB, where P-values are from one-way ANOVA tests.

## Results and discussions

### Effect of glucose on cell rolling

Our experiments mimic hyperglycemia by culturing neutrophils in glucose-rich media at three glucose concentrations (5, 13 and 25 mM) for 72 h. Neutrophil rolling experiments were carried out after this chronic glucose exposure at a range of physiologically relevant shear stresses (0.17–1.24 Pa) to determine the effects of hyperglycemia on cell rolling.

**Fig. 1:**
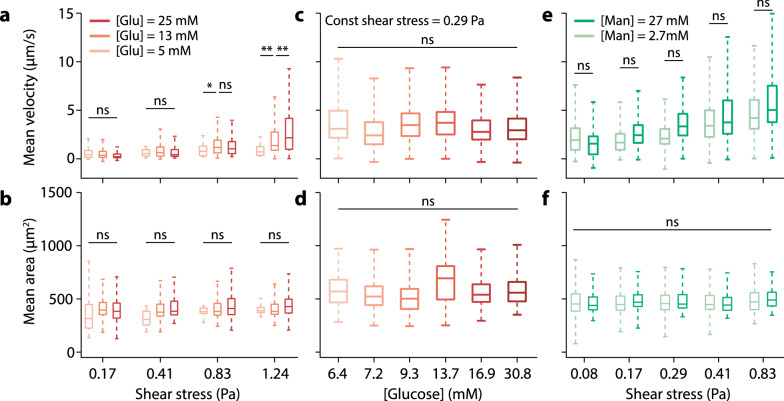
Effects of hyperglycemia on neutrophil rolling adhesion. **a** Neutrophil rolling velocities increase as a function of shear stress under different glucose concentrations (chronic exposure, dark to light red represent decreasing glucose concentrations). Error bars represent the first and third quartiles. **b** Cell projection area is not changed as a function of media glucose concentration. (n = 50, 655, 269, 16, 539, 105, 29, 335, 163, 33, 315, 134 cells) **c**, **d** Acute changes in glucose concentration have no detectable effect on neutrophil rolling velocity (c) or size (d). All data correspond to rolling at 0.29 Pa. (n = 432, 1213, 535, 326, 1787, 513 cells) **e**, **f** Neutrophils cultured under the same glucose concentration but differing mannitol concentrations in media exhibit similar rolling velocity and size. (dark and light green represent high and low mannitol concentration. n = 1332, 474, 639, 751, 519, 391, 1100, 384, 231, 216 cells) Statistical significance was tested by 1-way ANOVA, (ns) no significance, (*) p-value < 0.01, (**) p-value < 0.001

As expected, we observed an increase in mean cell rolling velocity with increasing shear stress [20]. This result holds at all 3 glucose concentrations tested. At low shear stress (0.17 and 0.41 Pa), we observe no statistical significance (p-values > 0.05) in cell rolling velocity at different glucose concentrations (Fig. 1a). However, at 0.83 Pa shear stress, we observed a statistically significant increase in rolling velocity from normal (5 mM) to hyperglycemic (13 and 25 mM) conditions, although the difference between 13 and 25 mM is not statistically significant (Fig. 1a). At the highest shear stress (1.24 Pa), there is a substantial increase in rolling velocity at higher glucose concentrations (Fig. 1a). This increase in neutrophil rolling velocity resulting from chronic hyperglycemic growth conditions may have immunological consequences in vivo*,* such as increasing the difficulty of extravasation into tissues which requires a slowing of cell rolling prior to rolling arrest at the target site.

We hypothesize two possible mechanisms that could lead to an increase in rolling velocity under hyperglycemic exposure: 1. A decrease in PSGL-1 expression on the neutrophil surface leads to weaker adhesive interactions with the P-selectin-coated surface, and 2. An increase in cell size where shear flow exerts a greater force to move the cells. However, we observed no statistically significant change in cell sizes among the test conditions (Fig. 1b). This indicates that glucose alone is not changing the phenotypic size of the cell within the chronic incubation periods. Furthermore, cells maintain the same size over an extensive range of shear stresses, indicating that the cells remain rigid and cell deformation from shear stress is insufficient to explain the observed velocity changes.

Next, we examine whether the effect of glucose on cell rolling velocity requires chronic (72 h) or acute (< 1 h) glucose exposure. For comparison, the doubling time for HL-60 cells is 36–48 h, and differentiation takes a minimum of 12 h after DMSO exposure [16]. We mimicked the effects of acute hyperglycemia exposure on neutrophils by exposing cells cultured under normoglycemic conditions to various glucose concentrations at the time of rolling experiments only. Our results indicate that neither the cell rolling velocity (Fig. 1c) nor cell size (Fig. 1d) is affected by acute glucose exposure over a wide range (6.4–30.8 mM) of glucose concentrations. This result shows that chronic glucose exposure is necessary for changing the neutrophil rolling behaviour.

Lastly, we examine whether chronic exposure to a non-metabolic sugar, mannitol, induces similar effects as glucose. Exposure to mannitol also serves as a control for osmotic pressure caused by changes in sugar concentration. We replaced glucose with mannitol under the same cell culturing and rolling conditions. The cell rolling velocity increased only slightly (< 10%) at high mannitol concentration (Fig. 1e) compared to an increase of up to 400% in the case of glucose (Fig. 1a). The change in rolling velocity at high vs low mannitol concentration was not statistically significant at any of the shear stresses recorded. Furthermore, different concentrations of mannitol induced no change in the cell size across the whole range of shear stress (Fig. 1f). We observed that cell rolling speed in mannitol was higher than in glucose (Fig. 1a vs. 1e). At high shear stress (1.24 Pa), most cells detached from the surface and were unable to sustain rolling under the mannitol condition, indicating that mannitol may impede the binding of P-selectin to PSGL-1.

Overall, glucose specifically increased the rolling velocity of neutrophils while cell size was unaffected. Since our assay is specific to P-selectin:PSGL-1 interactions, the results suggest changes to PSGL-1 as the main culprit of the observed changes. However, it is difficult to assess whether the protein expression level and/or the glycosylation changes led to this functional change. Such change may result from an increased growth rate under high glucose concentration. Indeed, the cell growth rate increases with glucose in the culturing media, which is particularly true when considering that the insulin-resistant glucose transporter GLUT1 is expressed in neutrophils [17].

### Effect of pro-inflammatory cytokines on cell rolling

**Fig. 2:**
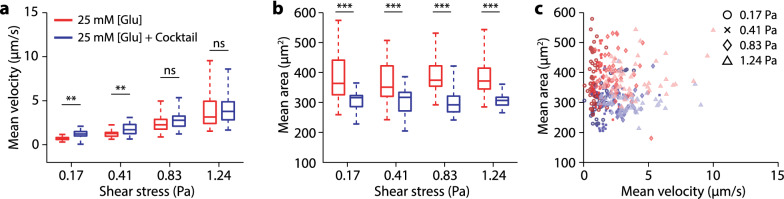
Effects of pro-inflammatory conditions on neutrophil rolling adhesion. **a** Neutrophil rolling velocity as a function of shear stress under hyperglycemic (red) and diabetic cocktail (blue) conditions. (n = 66, 50, 48, 50, 39, 42, 35, 28 cells) **b** Neutrophil cell projection area as a function of shear stress under hyperglycemic (red) and diabetic cocktail (blue) conditions. **c** Mean cell area vs. mean rolling velocity at different shear stresses (0.17 Pa–1.24 Pa) shows two clusters, indicating cells chronically exposed to diabetic cocktail are smaller and roll faster. Statistical significance was tested by 1-way ANOVA, (ns) no significance, (*) p-value < 0.01, (**) p-value < 0.001, (***) p-value < 0.0001

Cells chronically cultured in media containing a cocktail of factors found in T2D (hyperglycemic plus TNF-α, IL-6 and insulin, Table 1) were compared to those cultured under only hyperglycemic conditions in rolling experiments. These factors were chosen due to their pro-inflammatory effects on T2D [16–18]. We observed a significant increase (p-value < 0.001) in cell rolling velocity over the low shear stress regimes (Fig. 2a), corresponding to the physiological shear stress in veins. In contrast, the cell rolling velocity differences at high shear stresses are not statistically significant. However, we observe a statistically significant decrease in cell projection area (up to 25%) upon chronic exposure to the diabetic cocktail compared to hyperglycemic exposure alone (Fig. 2b).

**Table 1:** Cell growth conditions

Test group	Glucose concentration (mM)	IL-6 concentration (ng/mL)	TNF-α concentration (ng/mL)	Insulin concentration (nM)
Hyperglycemic	25	–	–	–
Diabetic Cocktail	25	10	10	1.0

The changes in cell size and rolling velocity under diabetic conditions are as expected in the presence of the pro-inflammatory cytokines TNF-α and IL-6. It has been shown that under diabetic conditions, the presence of excess cytokines leads to neutrophils exhibiting an activated phenotype typical of an inflammatory response [18, 19]. Neutrophil activation can lead to decreased expression of PSGL-1 and interaction with P-selectin [20]. Upon this weakened neutrophil-endothelial adhesion, there would be an increase in cell rolling velocity compared to normal physiological conditions. In addition, the presence of TNF-α promotes neutrophils to enter a caspase-mediated apoptotic pathway that can result in cell shrinkage [21]. These physiological changes due to diabetic conditions support the observed behavior of faster rolling velocity and decreased cell size.

When plotting the cell projection area against mean rolling velocity (Fig. 2c), two clusters corresponding to hyperglycemic and diabetic cocktail conditions emerge. Smaller cells generally have lower rolling velocities due to lower force from the shear flow. Surprisingly, the smaller neutrophil under diabetic cocktail conditions rolled faster than the larger cells cultured under hyperglycemic conditions. Because of our surface passivation and functionalization, cell rolling is explicitly supported by P-selectin:PSGL-1 interactions. For smaller cells to roll faster, a further decrease of PSGL-1 function on the neutrophil surface is required. Even at the higher shear stresses where the cell rolling velocity is similar, this holds as the cell size reduction was significant. This is also supported by the need for chronic exposure to hyperglycemia and the lack of such effect upon acute exposure, which does not allow the time to affect surface protein expression.

Interestingly, the increase in cell rolling velocity is inconsistent with previous reports showing decreased velocity in cells collected from diabetic patients [7]. The study differed in using HUVECs as a substrate instead of an engineered surface. HUVECs present many adhesion receptors that could interact with leukocytes and affect rolling. In addition, HUVEC surface function might be modulated by diabetic buffer conditions, making it difficult to separate the effect on rolling due to leukocyte alone. These uncertainties are not present in our study, as we exclusively investigate rolling via the P-selectin:PSGL-1 interaction.

## Conclusion

In conclusion, we observe a statistically significant increase in the rolling velocity of HL-60 derived neutrophils on a P-selectin surface due to chronic exposure to hyperglycemic conditions in growth media. This increase is independent of cell size and any osmotic effects caused by elevated sugar concentrations. We also observe a statistically significant decrease in cell size due to exposure to pro-inflammatory mediators. In contrast, the smaller neutrophils roll at a similar or faster velocity than those without the pro-inflammatory mediators. These observations lead us to conclude that chronic exposure of neutrophils to T2D conditions decreases PSGL-1 function, resulting in faster cell rolling and potentially less effective recruitment to target sites.

## Limitations

While the work examines, at a functional level, the effect of pro-inflammatory factors and hyperglycemic conditions on the rolling behaviour of leukocytes, we do not have the resources to perform proteomic studies to examine the mechanism behind our observed differences. Hence, we can only conclude that PSGL-1 function in rolling adhesion has been hampered under chronic T2D exposure. There are a few technical limitations: (1) because cells consume glucose, the glucose level we reported is an average concentration over 48 h, as we cannot keep the concentration constant. (We observe the glucose concentration drop up to 20% over 48 h). (2) According to the literature, up to 20% of HL-60 cells do not differentiate into neutrophils, which we cannot eliminate to produce a pure neutrophil population for the study.

## Data Availability

The datasets used in the current study are available from the corresponding author on request.

## Abbreviations

PSGL-1: P-selectin glycoprotein ligand 1
TNF-α: Tumour necrosis factor α
IL: Interleukin
T2D: Type 2 diabetes mellitus
HUVECs: Human umbilical vein endothelial cells
DMSO: Dimethyl sulfoxide
